# The inflammatory function of human IgA

**DOI:** 10.1007/s00018-018-2976-8

**Published:** 2018-11-29

**Authors:** Ivo S. Hansen, Dominique L. P. Baeten, Jeroen den Dunnen

**Affiliations:** 1 Amsterdam Rheumatology and immunology Center, Academic Medical Center (AMC), Amsterdam, The Netherlands; 2 Department of Experimental Immunology, Amsterdam Infection & Immunity Institute, Amsterdam UMC, University of Amsterdam, Meibergdreef 9, Amsterdam, The Netherlands

**Keywords:** FcαRI, Myeloid cells, Cytokines, Inflammation, Autoimmunity

## Abstract

The prevailing concept regarding the immunological function of immunoglobulin A (IgA) is that it binds to and neutralizes pathogens to prevent infection at mucosal sites of the body. However, recently, it has become clear that in humans IgA is also able to actively contribute to the initiation of inflammation, both at mucosal and non-mucosal sites. This additional function of IgA is initiated by the formation of immune complexes, which trigger Fc alpha Receptor I (FcαRI) to synergize with various other receptors to amplify inflammatory responses. Recent findings have demonstrated that co-stimulation of FcαRI strongly affects pro-inflammatory cytokine production by various myeloid cells, including different dendritic cell subsets, macrophages, monocytes, and Kupffer cells. FcαRI-induced inflammation plays a crucial role in orchestrating human host defense against pathogens, as well as the generation of tissue-specific immunity. In addition, FcαRI-induced inflammation is suggested to be involved in the pathogenesis of various chronic inflammatory disorders, including inflammatory bowel disease, celiac disease, and rheumatoid arthritis. Combined, IgA-induced inflammation may be used to either promote inflammatory responses, e.g. in the context of cancer therapy, but may also provide new therapeutic targets to counteract chronic inflammation in the context of various chronic inflammatory disorders.

## Introduction

Antibodies are an integral part of the human immune system. Of the five different classes of antibodies that are found in humans (IgA, IgD, IgE, IgG, and IgM), IgA is by far the most produced antibody in the human body, even surpassing all other classes combined [1]. Most of the IgA is present at mucosal sites, where it is produced as a dimer by locally residing plasma cells. IgA is also the second most abundant isotype in serum, where it is normally present at concentrations of 1–3 mg/mL. In circulation, IgA is generally found as a monomer, which is produced by plasma cells located in the bone marrow.

**Fig. 1:**
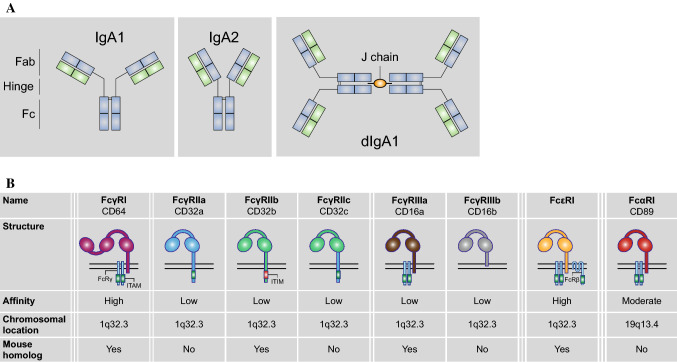
The human Fc receptor family. **a** IgA molecules consist of two domains, which are linked by a hinge region. IgA2 molecules have a shorter hinge region than IgA1, leading to a more Y-shaped conformation. The antigen-binding domain (Fab) binds to antigens, while the crystallizable fragment (Fc) domain can be recognized by Fc receptors. Furthermore, one molecule is made up of two identical heavy chains (in blue) and two identical light chains (in green). IgA molecules can be expressed as dimers when the Fc domains are connected to each other by a joining (J) chain. **b** Human FcRs are divided according to their binding capability to antibody subtype, FcγR, FcεR, and FcαR. FcγRs can be further subdivided into three types: FcγRI, FcγRII, and FcγRIII, which can be grouped based on their binding affinity to IgG (with FcγRI being the only high-affinity receptor). FcαRI is genetically located on a distinct location apart from the other receptors. The human FcR family differs quite significantly from the mouse FcR family

There are two IgA subtypes found in humans, IgA1 and IgA2. The subtypes differ at various sites in the heavy chain, however the most notable difference is found in the hinge region where IgA2 lacks 13 amino acids compared to IgA1 (see Fig. 1a for a schematic overview). Truncation of the hinge region in IgA2 leads to a reduced susceptibility to IgA1 bacterial proteases, which possibly explains the higher prevalence of IgA2 in mucosal secretions.

The main function of IgA has long been considered to be mostly ‘passive’ or anti-inflammatory. However, recently IgA has emerged as an inducer of ‘active’ immunity by controlling cytokine and chemokine production. In this review, we will briefly mention the passive function of IgA, but will subsequently focus on the inflammatory function of IgA in humans in the context of health and disease.

## Passive immunity by IgA: immune exclusion, neutralization, and antigen excretion

The most well-known function of IgA is that it provides passive immunity, through immune exclusion, pathogen neutralization, and antigen excretion, particularly at mucosal sites such as the gastrointestinal tract. In the intestine, IgA is produced in large quantities in dimeric form by plasma cells in the lamina propria, which contain the joining J chain that allows transportation over the epithelium by the poly Ig receptor and excretion into the lumen as secretory IgA (SIgA) [2, 3]. SIgA binds to both bacteria as well as bacterial products and is thereby able to prevent their interaction with the epithelium. Locally produced IgA by plasma cells in the lamina propria of the intestine is tailored to recognize the microbiota present in the lumen and particularly targets pathogenic bacteria [4]. During transport of IgA through the intestinal epithelial cells it is already able to bind to its target, which both facilitates excretion of antigens back into the lumen that have reached the lamina propria, but also neutralizes intracellular pathogens in the epithelial cells [5]. These functions of IgA have been called passive immunity and have long been thought to be the main role of IgA. Although this function of IgA is very important for homeostasis, several excellent reviews [5–7] cover this subject in detail and we will not further discuss it here.

## Active immunity by IgA: amplification or inhibition of cytokine production

In addition to the well-known passive functions, it has more recently become clear that IgA can also actively control immune responses. This immune activating function of IgA is effectuated by modulating the production of various key cytokines such as TNF and IL-1β by human myeloid immune cells, which is a pivotal step in controlling local and systemic immunity. In this regard, IgA has a dual role, as it can induce both inflammatory and immunosuppressive responses. Crucial for the active immune function of IgA is the binding to its receptor. Although different IgA receptors have been described (see Box 1 for an overview), the main IgA receptor that has been identified to be responsible for IgA-induced cytokine responses appears to be Fc alpha receptor (FcαRI; also known as CD89) [8]. FcαRI is one of the members of the family of Fc receptors, although it has some key distinctions which sets it apart from, e.g. the Fcγ receptors (recognizing IgG) or Fcε receptor (recognizing IgE) (Fig. 1b). FcαRI expression is restricted to the myeloid immune cell compartment and has been identified on neutrophils, monocytes, eosinophils, macrophages, and particular subsets of DCs such as intestinal CD103^+^ DCs and monocyte-derived DCs [8–13]. FcαRI does not contain any signaling motives in its cytoplasmic tail, but instead FcαRI relays signaling by association with the Fc receptor gamma chain (FcRγ), which contains an immunoreceptor tyrosine-based activation motifs (ITAM). Previously, FcαRI activation has been shown to lead to a variety of immune processes including degranulation, phagocytosis, chemotaxis, and antibody-dependent cellular cytotoxicity (ADCC) [14]. In contrast, FcαRI has long been considered to be a very poor inducer of cytokines by immune cells. Interestingly, FcαRI has no direct homolog in mice [15, 16], and since most of our knowledge on FcRs comes from mouse studies, this may partly explain why FcαRI-induced control of cytokine production has so long been underexposed.

Control of cytokine expression by antigen-presenting cells (APC) is essential for controlling inflammation and inducing both innate and adaptive responses [17, 18]. Generally, cytokine production is induced by APCs upon recognition of components of pathogens by several families of receptors, collectively known as pattern recognition receptors (PRR). These include the Toll-like receptors (TLR) [19], NOD-like receptors (NLR) [20], C-type lectin receptors (CLR) [21], and RIG-I-like receptors [22]. However, the immune response by APCs is not determined by stimulation of a single receptor, but rather a cooperation of multiple receptors [23–26].

A key feature of IgA-induced cytokine production is that FcαRI stimulation does not elicit cytokine production when stimulated individually, but that FcαRI collaborates with other receptors (mostly PRRs) to amplify or inhibit the production of specific cytokines. Notably, the ultimate FcαRI-induced cytokine profile is not uniform, but instead appears to be tailored to the immunological context, which depends on (1) the receptor that FcαRI interacts with, (2) the cell type involved, and (3) whether IgA binds to FcαRI in soluble or aggregated form. Below, we will discuss the role of FcαRI and IgA in regulating cytokine production in various tissues as well as its relevance to understanding and potential treatment of human diseases.

## Inhibitory signaling by IgA

The immunosuppressive function of monomeric or dimeric IgA has been known for decades [27]. Yet, the molecular mechanism behind this has long been unclear. In recent years, it has been described that FcαRI-induced ITAM signaling (which originally was only considered to promote inflammatory responses) can also negatively control inflammatory responses [28, 29]. This anti-inflammatory function of ITAMs has been named inhibitory ITAM (ITAMi), and has also been described for other receptors such as FcγRIIa and FcγRIII [30, 31]. Due to the low affinity of IgA monomers and dimers for FcαRI, circulating and unbound IgA bind only transiently, which results in ITAMi signaling under homeostatic conditions. This steady-state inhibitory signaling results in inhibition of several inflammatory processes, such as oxidative burst activity, chemotaxis and IgG Fc receptor mediated phagocytosis, as well as cytokine production [32–35]. For example, Pasquier et al. identified that stimulation of FcαRI with soluble IgA inhibits FcεRI-induced degranulation of mast cells, which prevents IgE-mediated asthma in transgenic mice expressing human FcαRI on myeloid cells [28]. In addition, Olas et al. showed that serum IgA suppresses the production of pro-inflammatory cytokines such as TNF and IL-6 from LPS-stimulated monocytes and PBMCs [36]. Mechanistically, the binding of monomeric or dimeric IgA is unable to cross-link FcαRI, causing transient Syk recruitment followed by recruitment of Src homology region 2 domain-containing phosphatase-1 (SHP-1) to the Fc receptor gamma chain (FcRγ). FcαRI and SHP-1 are then recruited to lipid rafts where both activating and inhibitory receptors are present, in a cluster known as an inhibisome. Due to the heterologous nature of these inhibisomes, activating signals are inhibited by the recruited SHP-1 resulting in inhibitory signaling [28, 37]. In summary, inhibition through FcαRI by monomeric or dimeric IgA plays an active role in homeostasis by suppression of inflammatory functions via ITAMi signaling.

## IgA-induced inflammation in host defense

Although FcαRI suppresses pro-inflammatory cytokine production under homeostatic conditions by ITAMi signaling, it also plays a crucial role in promoting inflammation during infection. The key to this switch from immune suppression to inflammation by FcαRI lies in the formation of IgA immune complexes. While under homeostatic conditions mainly monomeric and soluble IgA is present, IgA immune complex formation occurs when IgA aggregates are formed, e.g. when invading bacteria become opsonized with IgA, during secondary infection or by cross-reactivity of antibodies to pathogen structures [38–40]. FcαRI binds monomeric and dimeric IgA with moderate affinity (*K*a = ~ 10^6^/M), while IgA immune complexes bind avidly to FcαRI [14, 41], which upon binding induces classic ITAM signaling. Although IgA immune complexes can directly activate effector functions such as phagocytosis and degranulation [8], individual FcαRI stimulation does not directly induce cytokine production. Instead, FcαRI strongly amplifies inflammatory responses through collaboration with PRRs. As such, IgA immune complex formation functions as a danger signal that promotes inflammation in different tissues, which we will discuss below.

## IgA-induced inflammation in the intestine

In the intestine, IgA is produced in vast quantities, where it provides both passive and active immunity. Yet, it is important to realize that in the intestine the active and passive immune functions of IgA appear to be spatiotemporally separated. In the lumen, SIgA continually provides passive immunity through immune exclusion of commensal bacteria. In contrast, in the lamina propria immune complexes of conventional IgA generate active immunity only upon infection by eliciting pro-inflammatory cytokine production by immune cells, which is a crucial feature for induction of protective immunity in the intestine.

In most tissues, the recognition of pathogens by innate immune cells through PRRs directly leads to inflammatory responses. However, considering the extremely high amount of commensal microorganisms and their products, PRR activation is a steady-state phenomenon in the lamina propria of the gastrointestinal tract, which does not induce inflammation, but instead drives immune tolerance [42]. Therefore, the intestinal immune system requires a second signal to discriminate between homeostatic conditions and infection. Recently, it has been identified that IgA immune complex formation in the lamina propria functions as one of those second signals. Upon infection, bacteria that penetrate the epithelial layer are opsonized by local (dimeric) IgA, which is tailored to an individual’s microbiota and is mostly directed against colitogenic species [4, 39]. Remarkably, in the intestine only few FcαRI-expressing cells are present under homeostatic conditions to detect these IgA immune complexes, since intestinal macrophages lack FcαRI expression [13], and neutrophils and monocytes are mainly recruited after infection [43–45]. Instead, one of the main FcαRI expressing cells for the initial recognition of IgA immune complexes in the intestine are a subset of DCs, characterized by the expression of CD103 (αEβ7 integrin) [46]. While under steady-state conditions PRR stimulation of these CD103^+^ DCs by bacteria promote immune tolerance through activation of regulatory T cells [47, 48], co-activation of FcαRI by IgA-opsonized bacteria breaks the tolerance of human ex vivo and in vitro CD103^+^ DCs by strongly inducing key pro-inflammatory cytokines such as TNF, IL-1β, and IL-23. This FcαRI-PRR cross-talk on CD103^+^ DCs further promotes inflammation by activating T helper 17 (Th17) and intestinal type 3 innate lymphoid cells (ILC3) [46], which subsequently promotes neutrophil recruitment as well as tissue repair through IL-22 production [49, 50].

**Fig. 2:**
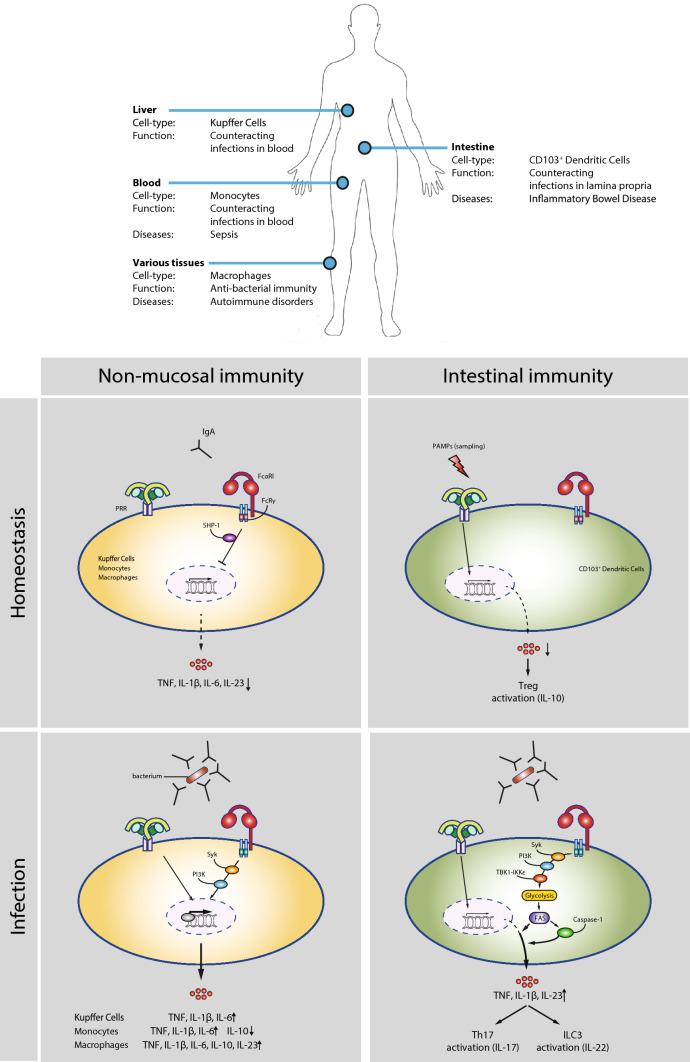
Tissue-specific FcαRI-mediated control of cytokine production in homeostasis and infection. FcαRI mediates both inflammatory and immunosuppressive responses in a cell-type and tissue-specific manner. FcαRI directs cytokine production under different conditions. Homeostasis: in non-mucosal tissues, monomeric IgA recognition by FcαRI during homeostasis leads to inhibition of pro-inflammatory cytokine production through ITAMi-mediated SHP-1 recruitment. In intestinal immunity, CD103^+^ DCs tolerogenic conditioning leads to activation through PRRs, which results into induction of regulatory T cells and IL-10 production. Infection: bacterial infection IgA immune complex formation is recognized by FcαRI leading to cross-talk with PRRs and pro-inflammatory cytokine production in Kupffer cells, monocytes, and macrophages through Syk and PI3K-mediated up-regulation of transcription resulting in distinct expression of cytokines. Upon intestinal infection, IgA opsonization provides a second signal through FcαRI activating Syk, PI3K, and TBK1-IKKε, which increases the glycolytic flux and fatty acid synthesis (FAS) that results in an inflammatory response mediated by increased mRNA translation and caspase-1 activation

Combined, IgA-induced inflammation is critically involved in counteracting bacterial infections in the intestine by functioning as a second signal that converts otherwise tolerogenic DCs into pro-inflammatory cells, thereby, controlling the delicate balance between tolerance and inflammation (Fig. 2).

## IgA-induced inflammation in non-mucosal tissues by serum IgA

IgA is strongly associated with mucosal immunity. However, IgA is also the second most common antibody isotype in serum, where it is primarily expressed as a monomer [1]. Similar to the non-secreted form in the lamina propria of the intestine, serum IgA in both monomeric and dimeric form can also induce pro-inflammatory cytokine production through activation of various FcαRI-expressing immune cells in different tissues, including liver, skin, and blood [51].

Invading pathogens that enter the bloodstream from the gastrointestinal tract will first be transported to the liver through the portal vein. Under homeostatic conditions, the resident macrophages in the liver, known as Kupffer cells, continually filter the blood from bacterial products originating from the intestine [52]. Under these basal conditions, Kupffer cells display immune tolerance to pathogen-derived molecules such as LPS [53, 54]. However, upon infection with intestinal bacteria, as frequently occurs in immunocompromised individuals [55–57], this tolerogenic response is converted to an inflammatory response. Kupffer cells highly express FcαRI, and it has long been known that IgA facilitates bacterial clearance in portal vein blood by Kupffer cells by promoting FcαRI-induced phagocytosis [12]. However, in addition to phagocytosis, FcαRI stimulation has recently also been shown to promote inflammation by Kupffer cells. Similar to the intestinal CD103^+^ DCs, the formation of serum IgA immune complexes by opsonization of bacteria breaks the tolerance of Kupffer cells to portal vein bacterial structures, resulting in production of pro-inflammatory cytokine production such as TNF, IL-1β, and IL-6 [51] (Fig. 2). These data indicate that in the liver IgA does not only mediate bacterial clearance, but that IgA also contributes to initiation of protective immunity by controlling the balance between immune tolerance and inflammation by Kupffer cells.

FcαRI is also expressed by other cytokine-producing myeloid immune cells such as monocytes and macrophages, situated in blood and tissues, respectively. Similar to Kupffer cells, serum IgA immune complexes enhance both phagocytosis and pro-inflammatory cytokine production by these cells, of which the latter function is again achieved through cross-talk with PRRs. For example, Hansen et al. showed that FcαRI stimulation with IgA immune complexes amplifies the PRR-induced production of pro-inflammatory cytokines such as TNF by human monocytes and macrophages [51]. However, importantly, the nature of the inflammatory response induced by IgA immune complex formation appears to be cell type-specific, since the ultimate cytokine profile induced by FcαRI-PRR cross-talk differs depending on the cell type involved (summarized in Fig. 2). For example, while serum IgA immune complexes strongly suppress the production of the anti-inflammatory cytokine IL-10 by monocytes, IL-10 production is increased by macrophages [51]. Although the mechanism behind these cell type-specific cytokine profiles are still unknown, the physiological function of this IgA-mediated effect is most likely to provide tissue-specific immune responses to invading pathogens.

While the cytokine profile induced by FcαRI-PRR cross-talk is cell-type specific, there is common ground in the amplification of Th17-promoting cytokines, such as IL-1β, IL-6, IL-23, and TNF [58, 59]. This suggests that serum IgA-induced inflammation, either in the intestine, liver, blood, or skin, is particularly important for counteracting extracellular pathogens, such as bacteria and fungi [60].

## Distinct molecular mechanisms of IgA-induced inflammation

Mechanistically, FcαRI activation by complexed IgA has thus far been identified to amplify cytokine responses on three different levels, i.e. by increasing (1) gene transcription, (2) gene translation, and (3) caspase-1 activation for post-translational amplification. Remarkably, the underlying molecular mechanism of FcαRI-induced cytokine production is cell-type specific. For example, FcαRI amplifies cytokine production in intestinal CD103^+^ DCs by strongly increasing gene translation (with no detectable effect on gene transcription) [46], while in Kupffer cells, monocytes, and macrophages FcαRI co-stimulation increases pro-inflammatory gene transcription [51]. Below we will discuss the mechanistic differences and similarities between the different cell-types (for a schematic overview see Fig. 2).

The mechanism behind FcαRI-TLR cross-talk is currently best understood in intestinal CD103^+^ DCs, where it was identified to depend on both amplified gene translation and caspase-1 activation. In the intestine, the continuous PRR activation by microbial structures under steady-state conditions induces pro-inflammatory cytokine gene transcription, but not gene translation, resulting in a net outcome of non-responsiveness or immune tolerance to intestinal microbiota. Yet, upon infection, IgA immune complexes activate FcαRI on CD103^+^ DCs, leading to activation of the kinases spleen tyrosine kinase (Syk) and phosphoinositide 3-kinase (PI3K), which are classical components of Fc receptor gamma chain (FcRγ) signaling [61, 62]. Subsequently, downstream FcαRI signaling through kinases TBK1 and IKKε ultimately results in amplification of both gene translation (of TLR-induced mRNA) and caspase-1 activation (for post-translational amplification).

Importantly, both FcαRI-induced gene translation and caspase-1 activation crucially depend on the induction of metabolic changes in CD103^+^ DCs. In recent years, it has become clear that for induction of pro-inflammatory responses these intracellular metabolic changes in immune cells are of crucial importance, which is collectively referred to as metabolic reprogramming [63]. Metabolic reprogramming is necessary to meet the high demands associated with inflammation, and is an essential process that occurs in various immune cells, including DCs, macrophages and T cells [64, 65]. While previously particularly PRRs were considered to induce metabolic reprogramming, it has recently been identified that also IgA, through FcαRI, is able to induce metabolic changes in DCs [46]. FcαRI activation strongly increases the glycolytic rate in CD103^+^ DCs, which drives de novo synthesis of fatty acids for the expansion of the endoplasmic reticulum, which then enables gene translation of pro-inflammatory cytokines such as TNF [46] (Fig. 2).

In contrast to most of the pro-inflammatory cytokines, FcαRI co-stimulation on CD103^+^ DCs does not enhance the gene translation of the pro-inflammatory cytokine IL-1β. Instead, IgA immune complexes amplify IL-1β production through caspase-1 activation, which is also dependent on FcαRI-induced metabolic reprogramming. IL-1β is generally produced after PRR stimulation as an inactive pre-cursor, known as pro-IL-1β, which has to be cleaved by caspase-1 into its bioactive form [66]. FcαRI activates caspase-1 most likely through Syk-induced glycolysis and fatty acid synthesis, similar to the pathway that is activated by caspase-1 activation by NLR family pyrin domain containing 3 (NLRP3) [67–69]. Thus, in CD103^+^ DCs, FcαRI-induced amplification of cytokine production at both the translational and post-translational level crucially depend on metabolic reprogramming.

Compared to intestinal CD103^+^ DCs, less is known about the molecular mechanisms of IgA-induced inflammation in liver, blood, and skin. Upstream signaling of FcαRI in Kupffer cells, monocytes, and macrophages is largely similar to CD103^+^ DCs in its dependence on Syk and PI3K [51]. However, the downstream mechanisms are clearly distinct, since in Kupffer cells, monocytes, and macrophages FcαRI co-stimulation amplifies cytokine gene transcription, instead of translation. The reason for distinct FcαRI signaling in intestinal CD103^+^ DCs is still speculative, but could be related to the unique immunological milieu in the intestine [70, 71]. Although FcαRI-dependent caspase-1 activation has not been studied in Kupffer cells, monocytes, and macrophages, this does seem likely to occur considering the discrepancy between IL-1β gene transcription and protein production [51]. Similarly, it is yet unknown whether FcαRI stimulation also induces metabolic reprogramming in these cells. While metabolic reprogramming is not commonly involved in affecting gene transcription [72], it could be responsible for caspase-1 activation in these cells, similar to the CD103^+^ DCs.

## IgA deficiency: compensation through IgG?

Considering the crucial function of IgA-induced passive and active immunity, it is remarkable that IgA deficiency has relatively mild effects and is in fact one of the most common primary immunodeficiency [73]. The absence of symptoms in IgA deficiency points towards a high level of redundancy. Regarding the passive immune function of SIgA, IgM is considered to compensate for the lack of IgA, since IgM can be transported to mucosal surfaces using the same polymeric IgR as IgA [74, 75]. In contrast, it is less clear how the human immune system can compensate for the capacity of IgA to induce cytokine production, since this is unlikely to also depend on IgM. Here, we propose that IgG may be important to compensate for this active immune function of IgA. IgG is present in the lamina propria of the intestine [76] and is expressed in serum in even higher concentrations than IgA [77]. Furthermore, most cytokine-producing FcαRI-expressing myeloid cells also express FcγRs, including intestinal CD103^+^ DCs, monocytes, and macrophages [78, 79]. In addition, cytokine production and subsequent immune cell activation is remarkably similar between FcαRI and FcγRs, since they both promote Th17 responses upon recognition of opsonized bacteria [80, 81]. In this regard, it is also interesting that IgA-deficient patients with concurrent deficiency of IgG2, the main IgG subclass directed against bacteria [82], are more symptomatic, which includes upper respiratory tract infections and diarrhea [83, 84]. Since FcγR-induced cytokine production has indeed been demonstrated for intestinal CD103^+^ DCs [46], monocytes [85, 86], and several macrophage subsets [87–89], it, therefore, seems likely that IgG can compensate for the loss of IgA in various tissues, including the lamina propria of the intestine, blood, and skin.

Remarkably, IgA deficiency is associated with several chronic inflammatory disorders, including rheumatoid arthritis, systemic lupus erythematosus, and celiac disease [75]. The reason for the association between IgA deficiency and these disorders is still largely unclear. One possibility is that low concentrations of soluble IgA result in less ITAMi-dependent immune suppression under homeostatic conditions, thereby lowering the threshold for immune activation. In contrast to this lack of soluble IgA, the presence of IgA immune complexes can also contribute to the development of these chronic inflammatory disorders, as discussed in the next paragraph.

## IgA-induced inflammation in inflammatory diseases

Although the physiological function of IgA-induced inflammation is to provide host defense by counteracting bacterial infections, undesired or excessive activation of this mechanism may lead to pathology by contributing to the development of chronic inflammation. IgA-induced inflammation could worsen pathology in IBD patients, since the damaged epithelium leads to massive presence of IgA immune complexes (from opsonized commensal bacteria) in the lamina propria. In addition, several autoimmune disorders, such as RA, SLE and celiac disease, are characterized by the presence of IgA autoantibodies. These autoantibodies can form immune complexes, thereby undesirably instigating local and/or systemic inflammation. Next, we will discuss FcαRI-mediated inflammation in the context of various chronic inflammatory disorders.

## Inflammatory bowel disease

IBD is a chronic relapsing disorder of the intestinal tract which is characterized by gastrointestinal inflammation and disruption of the epithelium. IBD encompasses all inflammatory bowel disorders, of which there are two major forms, Crohn’s disease and ulcerative colitis [90]. These disorders differ in both clinical as well as pathological features, suggesting distinct pathogeneses. However, already decades ago it has been described that altered cytokine expression by immune cells in the intestinal lamina propria is associated with inflammation in both IBD forms [91, 92].

IBD patients are characterized by impaired barrier function of the intestinal epithelial layer, resulting in influx of IgA-opsonized bacteria. As a result of the presence of these IgA immune complexes in the lamina propria, FcαRI-induced inflammation is very likely to be chronically activated in IBD patients. Indeed, the cytokines that are amplified by FcαRI cross-talk in the intestine are also strongly associated with the pathogenesis of IBD. TNF plays a central role in IBD pathogenesis, which is evident by the current use of TNF inhibition for as a standard therapy for both Crohn’s disease and ulcerative colitis [93, 94]. In addition, both IL-1β and IL-23 are implicated in the pathogenesis of Crohn’s disease, with anti-IL-23 antibodies as an important candidate for therapeutic use [95].

Although IgA-induced inflammation is very likely to occur in the intestine of IBD patients, it is less clear how it affects the pathogenesis of the disease. Theoretically, FcαRI-induced inflammation could play a role in IBD pathogenesis by either being overactive, or by being impaired. On the one hand, considering the powerful pro-inflammatory response induced by IgA-induced inflammation, a predisposition of individuals for prolonged and/or excessive activation of this mechanism could lead to extensive collateral damage, and as such contribute to IBD pathogenesis. But on the other hand, impaired IgA-induced inflammation could lead to chronic inflammation as well. The main physiological function of IgA-induced inflammation is to provide protective immunity against invading pathogens, and, therefore, impaired functionality could hamper the orchestration of intestinal anti-bacterial immune responses, which eventually could also lead to chronic bacterial infection and inflammation. In this regard, it is important to note that FcαRI stimulation of intestinal DCs does not only induce inflammation, but also stimulates tissue repair in the intestine, by strongly increasing the production of IL-22 by ILC3 [46].

Thus, while IgA-induced inflammation is active in inflamed tissue of IBD patients, it is still unclear whether it contributes to IBD pathogenesis by either being overactive (leading to excessive activation), or by being impaired (leading to insufficient host defense against bacteria, ultimately also resulting in chronic infection and inflammation).

## Celiac disease

Celiac disease is an inflammatory disorder of the small intestine, which is characterized by presence of autoantibodies and T cell-mediated destruction. In celiac disease patients, exposure to the dietary antigen gluten is the causative pathological factor [96, 97]. Exposure to gluten leads to lesions in the small intestine characterized by villous blunting, epithelial crypt cell hyperplasia and leukocyte infiltration including plasmacytosis in the lamina propria [97].

In addition to antibodies against gluten, celiac disease patients express high levels of autoantibodies against the enzyme transglutaminase 2 [98], which is produced locally in the mucosa of the intestine [99]. On average 10% of plasma cells in a disease lesion are directed against transglutaminase 2 and, importantly, most of these plasma cells produce antibodies of the IgA isotype [100]. The abundant presence of the autoantigen, leading to the formation of IgA immune complexes, in combination with commensal bacteria that can enter via the lesions is, therefore, very likely to promote inflammation through FcαRI-PRR cross-talk. Hence, FcαRI-induced inflammation by IgA autoantibodies are likely to worsen the pathology in patients suffering from celiac disease.

## Rheumatoid arthritis

RA is a chronic autoimmune disease occurring in 1% of the population and is characterized by inflammation and damage of the joints. Although the pathogenesis is not fully understood, it is clear that pro-inflammatory cytokines such as TNF play a crucial role, which is underlined by the great clinical improvement after neutralization of these cytokines [101, 102]. One of the hallmarks of RA is the presence of autoantibodies. The most prominent of these autoantibodies present in RA patients are anti-citrullinated protein antibodies (ACPA) and rheumatoid factor (RF), which are already present long before the onset of disease [103, 104]. ACPA recognize citrullinated extracellular matrix proteins in the joint, while RF binds to the Fc part of IgG antibodies. The binding of these autoantibodies to their antigens leads to the formation of large insoluble immune complexes [105], which in turn enable their recognition by Fc receptors.

The autoantibodies present in RA patients can be of various isotypes, including IgA [106]. The presence of IgA rheumatoid factor (RF) has been recognized as a predictive marker for RA, leading to increased systemic cytokine production [106, 107]. Furthermore, ACPA immune complexes formed in the presence of IgA RF lead to increased expression of RA-associated cytokines [108]. Myeloid immune cells such as monocytes and macrophages that recognize these immune complexes are also the main cellular source of pathogenic pro-inflammatory cytokines such as TNF, IL-1β, and IL-6. However, similar to its function in host defense against bacteria, FcαRI essentially needs to collaborate with PRRs to induce cytokine production. In RA synovia, PRR activation is not induced by microbial compounds, but by endogenous ligands that are present as a result of tissue damage and cell death, and which are generally referred to as damage-associated molecular patterns (DAMPs) [109]. Combined, IgA immune complexes and PRR ligands particularly drive the production of RA-associated pro-inflammatory cytokines by myeloid immune cells [51, 81].

Taken together, FcαRI, particularly in synergy with PRRs, may contribute to RA pathogenesis by promoting pro-inflammatory cytokine production in response to IgA autoantibodies, similar to the more established pathogenic function of FcγRs [110–113].

## Other autoimmune diseases: IgA nephropathy, linear IgA bullous disease, and dermatitis herpetiformis

In addition to aforementioned diseases, there are various other disorders that are characterized by the presence of autoantibodies of the IgA isotype. In principle, FcαRI-PRR cross-talk will occur in any disorder in which the combination of IgA immune complexes, (endogenous or microbial) PRR ligands, and FcαRI/PRR expressing immune cells are present. Although at this time there is still little direct evidence, several diseases are likely to fulfil these criteria. An important example is IgA nephropathy, the most prevalent form of primary glomerulonephritis that often leads to end-stage kidney failure. In IgA nephropathy patients, IgA immune complexes accumulate in the glomerular mesengium [114]. These IgA immune complexes promote macrophage infiltration and induce the release of pro-inflammatory cytokines in FcαRI transgenic mice, which interestingly is not only dependent on FcαRI but also on transferrin receptor I [115, 116].

IgA immune complexes may also play a role in skin blistering diseases such as linear IgA bullous disease and dermatitis herpetiformis. Linear IgA bullous disease patients are characterized by expression of IgA autoantibodies against collagen XVII, a transmembrane protein involved in maintaining cell–matrix adhesion in the skin [117]. In addition to neutrophils that are recruited to the inflamed skin and are activated by FcαRI stimulation to induce tissue damage [118], IgA immune complexes may promote inflammation in a similar manner by eliciting FcαRI-induced pro-inflammatory cytokines. Dermatitis herpetiformis is clinically similar to linear IgA bullous disease, and is also characterized by the presence of IgA autoantibodies in the skin. Dermatitis herpetiformis is strongly associated with celiac disease [119] and, therefore, could induce IgA-induced inflammation in skin in a similar manner as celiac disease drives intestinal inflammation. Future studies are needed to establish whether IgA-induced pro-inflammatory cytokine production is indeed involved in the pathogeneses of these diseases.

## Therapeutic opportunities

As discussed above, the stimulation of FcαRI with IgA immune complexes, in cooperation with PRRs, promotes inflammatory cytokine responses. During infection, IgA-induced inflammation allows for a tailored immune response to counteract invading pathogens. However, when overactive or deficient, it may also lead to the development of chronic inflammation. Therefore, modification of IgA-induced cytokine responses may provide opportunities for therapeutic intervention, either by reducing or enhancing these inflammatory responses.

Intravenous immunoglobulin (IVIG) treatment has been used for several decades now to treat inflammatory disorders. Initially it was used as therapy for immunocompromised patients as a replacement therapy but was shown to have anti-inflammatory effects. IVIG is mostly based on the immunomodulatory function of IgG [120]. However, as discussed earlier, in its monomeric form IgA also has the ability to induce inhibitory signaling via FcαRI. In transgenic mice that express human FcαRI on monocytes/macrophages this leads to a reduction of arthritis via ITAMi signaling [121]. While IgA has been associated with immunosuppression, to date still very few studies have been performed with intravenous IgA. This is most likely a combination between the difficulty of purifying IgA, as well as the fast turn-over rate that IgA has in the systemic circulation, although Fc engineered IgA2 molecules have shown an improved pharmacokinetics in vivo [122, 123].

As an alternative to inducing immune suppression, it may be promising to specifically interfere with the downstream signaling that is responsible for FcαRI-TLR cross-talk. Signaling molecules such as Syk, PI3K and TBK1-IKKε have been identified as pivotal players in the induction of cytokines by FcαRI [46, 51]. Interestingly, disease activity in RA patients was significantly reduced by therapeutic inhibition of Syk using oral small-molecule inhibitor R788 [124]. In addition, Syk expression in intestinal DCs was shown to play an important role in inducing colitis in mice [125] indicating that inhibition of Syk could be helpful for IBD patients. Therapeutic inhibitors for PI3K are being developed as well, most for treatment in cancer, but some are also being tested for application in inflammatory disorders [126, 127]. It is yet unclear which form of PI3K is required for FcαRI-PRR cross-talk, and, therefore, narrowing this down could help with precisely targeting IgA-induced inflammation in chronic inflammatory disorders. Therapeutic TBK1-IKKε inhibitors Amlexanox and Rebamipide are already in clinical use for inflammatory disorders such as asthma and gastric ulcers [128, 129] and may be used to target IgA-induced inflammation. Additionally, FcαRI-TLR cross-talk was identified to depend fatty acid synthase (FAS) activity, for which inhibitors are now being developed and tested in the context of cancer treatment [130, 131]. When effective, inhibition of FAS could have therapeutic potential in inflammatory disorders as well. Further elucidation of the involved signaling pathways in FcαRI-TLR cross-talk may extend the repertoire of small-molecule inhibitors that can be used for the treatment of chronic inflammatory disorders.

While interfering with FcαRI-induced cytokine production may be beneficial in the treatment of autoimmune disorders, enhancing inflammation could provide new opportunities in the treatment of diseases such as cancer [132]. The local environment of solid tumors is dominated by the presence of myeloid derived suppressor cells, including the presence of anti-inflammatory M2 macrophages, which inhibit the generation of effective anti-tumor responses [133–135]. Since M2 macrophages express FcαRI, (co-)stimulation of these cells may convert these anti-inflammatory cells into pro-inflammatory cells, similar to the conversion of other tolerogenic immune cells such as Kupffer cells and intestinal DCs [46, 51]. In this regard, it is interesting that human M2 macrophages have previously been shown to be converted to pro-inflammatory cells upon stimulation with IgG immune complexes that activate FcγRs [89]. This is especially interesting since IgA has been shown to have a greater therapeutic potential in treating solid tumors than IgG, since IgA is far more effective in mediating recruitment of and killing by neutrophils [122, 123, 136–138]. Hence, the combination of IgA antibodies and an adjuvant such as a PRR agonist or IFNγ to promote local inflammation, as well as the direct killing of tumor cells, may be a useful tool in the treatment of solid tumors.

Taken together, understanding the underlying mechanisms and cell-type specific responses of IgA-induced cytokine production could provide new therapeutic opportunities for a large variety of disorders, including chronic inflammatory disorders, autoimmune diseases, and cancer.

## Concluding remarks

Here, we have discussed a novel property of IgA in inducing inflammation via FcαRI in various tissues by directing cytokine responses. Yet, it is important to realize that FcαRI also mediates various other immunological processes, including phagocytosis, degranulation, and ADCC. Notably, many of these processes will occur simultaneously, although sometimes by different cell types. Therefore, in view of therapeutic opportunities, it would be useful to specifically induce or interfere with one feature of FcαRI, while leaving other functions unharmed. In this regard, the recent findings that indicate that FcαRI signaling uses cell type-specific signaling pathways may provide opportunities to modulate IgA-induced cytokine responses in a tissue-specific, and perhaps even disease-specific, manner. However, to really exploit the targeting of distinct FcαRI functions, more knowledge about the underlying molecular mechanisms of the different FcαRI functions in different human immune cells is required.

## Box 1: Other IgA receptors

Several different receptors have been described to bind IgA. There are major differences in both affinity and function, which is summarized below.

- Poly Ig receptor is expressed on epithelial cells and is crucial for the transport of immunoglobulin to the lumen of mucosal tissues. The ligand for the PIgR is the J-chain and, therefore, it is able to bind both IgA and IgM. After binding, the receptor is internalized and transported across the cell, whereupon the immunoglobulin is released into the lumen [2, 3].
- Fc alpha/mu receptor is a receptor expressed on hematopoietic and non-hematopoietic cells, including B cells [139]. It binds both IgM and IgA but has a much higher affinity for IgM [140]. IgA binding likely occurs during complex formation and has been associated with removal of IgA complexes from circulation.
- Transferrin receptor expressed on mesangial cells, epithelial cells, and B cells, where it is able to bind IgA1, but not IgA2 [141]. Transferrin receptor has been implicated in the formation of IgA1 complexes in IgA nephropathy [142, 143].
- Asialoglycoprotein receptor is a C-type lectin receptor, expressed on hepatic cells. It recognizes and binds to terminal galactose or N-acetylgalactosamine residues. ASPGR has been shown to be involved in clearance of IgA from the circulation [144–146].

Other receptors, including FcRL4 [147], DC-SIGN/SIGNR1 [148], SC-receptor [149], and M-cell receptor [150] have been described to bind IgA, but the exact role the binding plays is not well defined yet.
